# Transcription factor c-Maf: a checkpoint that programs autoimmunity

**DOI:** 10.3389/fimmu.2025.1682098

**Published:** 2025-11-26

**Authors:** Na Liu, Jin Zhang, Pingping Wang, Shuyu Jin, Chunjuan Yang, Xinyi Yan, Jing Xu, Hui Wang, Wenchang Sun, Donghua Xu

**Affiliations:** 1 Center of Medical Research, Weifang People’s Hospital, Shandong Second Medical University, Weifang, Shandong, China; 2 Department of Rheumatology, Weifang People’s Hospital, Shandong Second Medical University, Weifang, Shandong, China; 3 Department of Gynecology and Obstetrics, Weifang Maternal and Child Health Hospital, Shandong Second Medical University, Weifang, Shandong, China; 4 School of Pharmacy, Shandong Second Medical University, Weifang, Shandong, China; 5 Department of Gynecology, Weifang People’s Hospital, Shandong Second Medical University, Weifang, Shandong, China

**Keywords:** autoimmunity, c-Maf, immune checkpoint, inflammation, autoimmune diseases

## Abstract

The transcription factor c-Maf, a member of the Maf family characterized by its basic domain and b-Zip DNA-binding motif, is a pivotal regulator of immune cells development and function. It governs immune cells growth, differentiation, function, and immune responses. This review explores the mechanistic role of c-Maf and its associated signaling networks in modulating autoimmunity and inflammation. We highlight its dual function as an immune checkpoint that suppresses pathological inflammation while promoting protective immunity, underscoring its therapeutic potential in autoimmune diseases.

## 1 Introduction

The Maf (Musculoaponeurotic fibrosarcoma) family originates from the AS42 virus, where it was first identified as a viral oncogene (1). Maf proteins are characterized by a conserved basic leucine zipper (b-Zip) motif, which mediates DNA binding via a leucine zipper structure that promotes homodimerization. This structural feature facilitates Maf proteins to recognize Maf recognition elements (MARE) and function as nuclear transcription factors (2). Maf family proteins can be categorized into two primary groups based on their molecular weight: large Maf proteins and small Maf proteins (3). As a large Maf transcription factor, c-Maf contains multiple functional domains, including an N-terminal acidic transactivation domain, a histidine/glycine repeat region, an extended homology domain, and a C-terminal b-Zip domain. The leucine zipper, evolutionarily conserved across species, facilitates dimerization with other b-Zip-containing transcription factors. c-Maf binds selectively to MARE and Maf half-sites that are enriched in 5-AT motifs, thereby regulating cellular processes such as proliferation, differentiation, and immune function.

As a transcription factor, c-Maf plays an oncogenic role in various cancers, which drives tumorigenesis through multiple mechanisms, including promoting cancer cell proliferation, adhesion and migration, angiogenesis and immune evasion (4–6). It also serves as a key regulator of intestinal cell differentiation and function, modulating intestinal villus division, nutrient absorption, and the maintenance of intestinal immunity and microbial homeostasis (7–9). Research has demonstrated that c-Maf plays a pivotal role in T and B lymphocytes differentiation and M2 polarization by regulating cytokine expression, particularly IL-4 and IL-10, which are essential for maintaining immune homeostasis (10). In inflammatory responses, c-Maf inhibits pro-inflammatory signaling pathways while promoting inflammation resolution through the modulation of anti-inflammatory mediators, such as IL-10. Furthermore, c-Maf is involved in the regulation of immune cells differentiation and function, contributing to immune balance and homeostasis. Dysregulation of c-Maf has been closely linked to chronic inflammation and autoimmune disorders. This review provides a comprehensive understanding of c-Maf in multiple immune disorders, underscoring the regulatory mechanisms and functions of c-Maf. Its mechanistic and functional contributions to the protective effects in autoimmune conditions provide future directions for the exploration of therapeutic applications of c-Maf in autoimmune diseases. Lastly, the review outlines prospective research avenues and practical implications for the integration of c-Maf into therapeutic strategies for autoimmune diseases.

## 2 The immune regulatory function of c-Maf

Accumulated studies have implicated that c-Maf is widely involved in the regulation of immune cells differentiation, function and the maintenance of tissue homeostasis. It plays critical roles in mediating adaptive immunity and innate immunity by regulating cytokine expression, metabolic reprogramming and epigenetic modifications. Increasing studies have revealed the regulatory network of c-Maf in immune cells, providing updated insight into understanding the pathogenesis of immune diseases and the exploration of new targeted therapies.

### 2.1 c-Maf and T cells

#### 2.1.1 c-Maf and CD8^+^ T cells

c-Maf is a key transcriptional regulator of CD8^+^ T cell function, with context-dependent roles in immune homeostasis and pathology. In skin-resident IL-17-producing CD8^+^ TRM (TRM17) cells, the ICOS-c-Maf-IL-7 axis promotes tissue residency and contributes to local inflammation control and tissue repair (11). Conversely, in the tumor microenvironment, IL-27-induced c-Maf cooperates with PRDM1 to drive the expression of co-inhibitory receptors, enhancing immunosuppressive signals, which helps prevent excessive immune activation (12–14). Besides, it also promotes CD8^+^ T cell dysfunction and exhaustion, facilitating tumor immune escape. Furthermore, c-Maf activates the caspase 6, increasing CD8^+^ T cells susceptibility to apoptosis (15). Thus, c-Maf exerts pleiotropic effects on CD8^+^ T cells via modulating their functional differentiation, immune suppression, and apoptotic sensitivity with outcomes shaped by specific physiological or disease settings.

#### 2.1.2 c-Maf and Tregs

c-Maf is a key transcription factor involved in the subset-specific differentiation and functional specialization of regulatory T cells (Tregs). Tregs themselves comprise multiple phenotypically and functionally distinct subsets, each shaped by specific lineage-defining transcription factors. For instance, RORγt^+^ Tregs are predominant in the colon and help restrain intestinal inflammation, whereas Bcl-6-expressing T follicular regulatory (Tfr) cells localize to lymphoid follicles and modulate antibody production by B cells (16). It has been shown that c-Maf is a key transcription factor driving the differentiation of Tregs subpopulations (17). In intestinal RORγt^+^ Tregs, c-Maf not only drives their terminal differentiation but also helps maintain gut homeostasis by promoting IL-10 secretion, curbing excessive PI3K/Akt/mTORC1 activation, and suppressing microbiota-induced Th17 cells responses and IgA production (18). c-Maf serves as a key transcription factor for host immune tolerance by driving the differentiation and function of inducible regulatory T cells (iTregs) to specifically suppress pathogenic Th17 cells (19). Meanwhile, the enhanced expression of c-Maf promotes IL-10 production in iTregs, thereby augmenting their immunosuppressive activity (20). Similarly, c-Maf is essential for the development and functional maturation of type 1 regulatory T (Tr1) cells, where it facilitates their characteristic IL-10 production (21–23). Thus, through its subset-specific roles, c-Maf fine-tunes Tregs differentiation and regulatory function across multiple tissue and immune contexts.

#### 2.1.3 c-Maf and Th2 cells

T helper 2 (Th2) cells are a specialized subset of CD4^+^ T cells involved in Th2-associated immunity through the secretion of key cytokines, including IL-4, IL-5, and IL-10 (24). The differentiation of Th2 cells is tightly regulated by the transcription factor c-Maf, which governs the initiation of Th2 cells differentiation and its function through complex mechanisms. Research indicates that naïve CD4^+^ T cells commence their differentiation in response to IL-4, with c-Maf serving as a Th2-specific transcription factor essential for CD25 expression during Th2 cells development (25). The regulation of c-Maf expression is orchestrated by various signaling pathways. IL-2 activates the STAT5 signaling pathway, which directly binds to specific promoter regions of the c-Maf gene to promote its expression (26). IL-6 increases c-Maf expression in TCR-activated T cells (26). STAT6 further integrates upstream signals, forming a regulatory cascade with GATA-3 and c-Maf (27). Furthermore, the post-translational modifications (PTMs) of c-Maf are vital for its functional activity. Specifically, tyrosine phosphorylation at residues Tyr21, Tyr92, and Tyr131 is essential for c-Maf’s recruitment to the IL-4 gene promoter, serving as a “molecular switch” for cytokine secretion (28). Additionally, SUMOylation at Lys33 represents a significant PTM event for c-Maf in Th cells, as it diminishes its transcriptional activity. Notably, the removal of the SUMO site does not affect the stability or localization of c-Maf but enhances its binding to IL-4 promoter (29).

Furthermore, the abnormal expression of c-Maf exerts influence beyond Th2 cells by activating nuclear factors such as NFATc1, which triggers endogenous IL-4 transcription in B cells and non-lymphoid cells, establishing localized immunoregulatory circuits (30). During Th2 cells activation, the rapidly upregulated SATB1 protein anchors the gene loci for IL-5, IL-4, and IL-13, facilitating the recruitment of GATA3, STAT6, and c-Maf to form transcriptional complexes (31). These complexes collaborate with chromatin remodeling factors to regulate cytokine expression. In addition to its direct role in Th2 cells differentiation, c-Maf plays a crucial role in maintaining Th1/Th2 cells homeostasis through dual mechanisms, including directly binding to the promoters of Th2 signature genes (e.g., IL-4) and indirectly suppressing Th1 cells-associated genes (e.g., IFN-γ) (32, 33). By influencing cytokine secretion and chromatin accessibility, c-Maf affects immune response. Overall, this study elucidates the multifaceted roles of c-Maf as an immunoregulatory hub, offering a foundation for transcription factor-targeted immune interventions.

#### 2.1.4 c-Maf and Th17 cells

T helper 17 (Th17) cells, a specialized subset of CD4^+^T cells that differentiate from naïve CD4^+^ T cells under the synergistic influence of IL-6, IL-21, TGF-β, and IL-23, are pivotal in the secretion of pro-inflammatory cytokines, such as IL-17 and IL-22. This process is mediated by the STAT3-mediated activation of RORγt, a transcriptional factor essential for the pathogenesis of autoimmune disorders (34). Recent studies have elucidated that the SRY-box transcription factor 5 (Sox5) interacts with c-Maf via the high mobility group (HMG) domain and the DNA-binding domain of c-Maf, thereby directly activating the RORγt promoter in CD4^+^ T cells to promote Th17 cells differentiation. However, c-Maf exhibits functional plasticity in Th17 cells. Under high concentrations of IL-6 and TGF-β, c-Maf binds the IL-22 promoter to inhibit its transcription (35). In inflammatory contexts, it is selectively upregulated to enhance the secretion of the anti-inflammatory cytokine IL-10 (36, 37). Importantly, c-Maf is indispensable for the development of intestinal regulatory Th17 cells, which attenuate effector T cells activity through IL-10 and co-inhibitory receptors, thus maintaining mucosal homeostasis (38). This dual role underscores c-Maf as an environmental sensor, balancing pro-inflammatory Th17 cells differentiation with tissue-specific anti-inflammatory responses. Such mechanistic insights highlight its potential as a therapeutic target for autoimmune diseases.

#### 2.1.5 c-Maf and Follicular Helper T Cells

Follicular helper T (Tfh) cells, a specialized subset of CD4^+^ T cells, migrate to germinal centers (GCs) within lymphoid follicles, where they interact with antigen-specific B cells to facilitate T cell-dependent antibody responses (39). Their differentiation is intensely regulated by a transcriptional network centered on B cell lymphoma 6 (Bcl6), which enhances the expression of chemokine receptors CCR7 and CXCR5, thereby guiding Tfh cells migration along CXCL13 gradients into the GCs. Notably, Tfh cells exhibit uniquely high c-Maf expression compared to other CD4^+^ subsets, underscoring its pivotal role in Tfh lineage commitment (40). Mechanistically, c-Maf activation by TGF-β drives CXCR5 and Bcl6 expressions, while chromatin remodeling and transcriptomic reprogramming further reinforce Tfh cells differentiation (41). This process is amplified by NF-κB (an upstream c-Maf regulator) and Thpok, which collaboratively establish a pro-differentiation transcriptional network (42, 43). Functional studies reveal that c-Maf and Bcl6 are co-expressed in early Tfh precursors, while the conditional deletion of c-Maf disrupts Tfh cells differentiation, GC B cells responses, and the production of high-affinity antibodies, highlighting its essential role in humoral immunity (44). Additionally, c-Maf governs the pre-Tfh to GC-Tfh via the Foxo1-Plekho1 axis, demonstrating its autonomous regulatory capacity over Tfh cells lineage commitment (45). Collectively, these findings demonstrate c-Maf as a central regulator of Tfh cells differentiation and T cell-dependent antibody responses (46).

While Th cell subsets employ distinct effector mechanisms, all subsets utilize IL-10 to mitigate excessive immune activation. c-Maf serves as a conserved regulator of IL-10 across subsets (e.g., Th2 cells, Th17 cells), influencing their differentiation, proliferation, and functions to maintain immune homeostasis and shape disease outcomes (32).

### 2.2 c-Maf and B Cells

B cells primarily mediate immune regulation through the presentation of antigens and the production of antibodies (47). Emerging evidence suggests that c-Maf negatively regulates B cells proliferation by influencing cellular metabolism, transporter activity, and mitochondrial proteins expression, ultimately impairing late-stage B cells differentiation and the formation of GC (48). Beyond its metabolic role, c-Maf also governs IL-10 expression in B cells. Upon LPS stimulation, c-Maf upregulation enhances its binding to the IL-10 promoter, driving dose-dependent IL-10 production (49–51). Recent studies highlight the critical role of regulatory B cells (Bregs), a B cell subset that produces IL-10 or TGF-β1, in maintaining immune tolerance by suppressing excessive inflammation (52). Bregs are pivotal in modulating chronic inflammatory diseases, such as colitis, rheumatoid arthritis, experimental autoimmune encephalomyelitis, and multiple sclerosis, as well as in infections and tumors (53). c-Maf signaling is essential for Bregs proliferation, with its deficiency leading to a notable decrease in pancreatic Bregs (54). Moreover, c-Maf regulates immunoglobulin-related genes and the production of tumor-specific antibodies (55). Therefore, c-Maf plays a vital role in balancing B cells homeostasis, integrating metabolic and cytokine signaling to balance activation and tolerance.

### 2.3 c-Maf and innate lymphoid cells

Innate lymphoid cells (ILCs) are a specialized subset of lymphocytes that function independently of T and B cells, playing vital roles in innate immune responses, tissue homeostasis, and infection recovery (56). ILCs are categorized into three primary groups, namely ILC1s, ILC2s, and ILC3s, based on their cytokine profiles, transcription factors expression, and surface receptor signatures (57). ILC1s are defined by transcription factor T-bet expression and produce pro-inflammatory cytokines, such as IFN-γ and TNF-α (58). ILC2s express GATA3 and drives type 2 inflammation by producing type 2 cytokines, such as IL-4, IL-5, IL-9, and IL-13 (59). ILC3s depend on RORγt and AHR for development and generate IL-17 and IL-22 to support mucosal immunity (60). In ILC2s, allergen exposure upregulates c-Maf, amplifying IL-5/IL-13 production and eosinophil recruitment. In contrast, c-Maf deficiency impairs type 2 cytokine and memory-like markers, highlighting its crucial effect for ILC2s function (61). Additionally, there is a specific subset of ILC2s that produce IL-10 (ILC2_10_s) in the lungs, where c-Maf-driven IL-10 suppresses ILC2s-mediated inflammation, mitigating lung pathology (62). In ILC3s, c-Maf acts as a negative regulator by directly inhibiting T-bet, preventing the conversion of ILC3s into ILC1s-like cells and thus preserving ILC3s stability (63, 64). Therefore, c-Maf serves as a multifunctional modulator of ILCs biology. It enhances type 2 responses in ILC2s, restrains inflammation via ILC2_10_s, and maintains ILC3s identity by antagonizing ILC1s plasticity. These findings highlight its potential as a therapeutic target for inflammatory diseases and immune dysregulation.

### 2.4 c-Maf and macrophages

**Figure 1:**
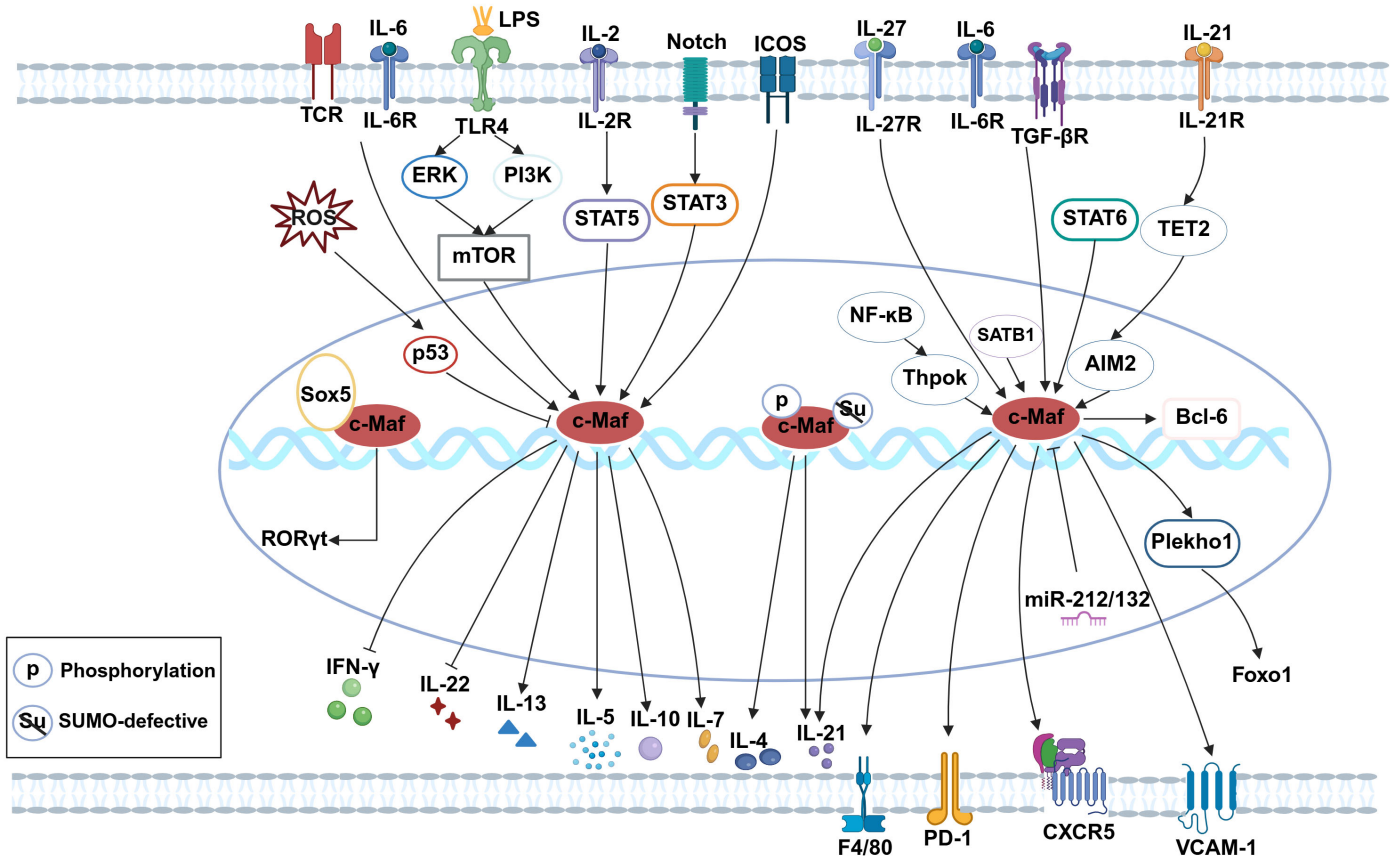
c-Maf-associated signaling pathways.

Macrophages are pivotal immune cells that maintain homeostasis through phagocytosis of pathogens and harmful debris. They are broadly classified into tissue-resident macrophages (self-renewing) and monocyte-derived macrophages (65). Tissue macrophages exhibit non-cancerous self-replication via the local proliferation of mature cells. Strikingly, transient reduction (66), inhibition (67), or depletion (68) of c-Maf confers stable self-renewal capacity to macrophages under steady-state conditions. c-Maf plays a crucial role in the differentiation of CD206^+^ lung interstitial macrophage subset (69). In c-Maf-deficient macrophages, the expressions of the tissue macrophage-specific receptor F4/80 (70), and VCAM-1 (71) are significantly decreased. By influencing the differentiation of perivascular macrophages (VAMs), c-Maf affects angiogenesis and metabolic syndrome (72). These results indicate that c-Maf not only influences macrophage types but also regulates their functional capabilities. When stimulated by LPS, c-Maf binds to IL-10 promoter to promote its production (73–75). In microglia, reactive oxygen species (ROS)-induced p53 activation suppresses c-Maf, exacerbating pro-inflammatory responses (76). Furthermore, c-Maf orchestrates M2-associated genes expressions, critically regulating tumor-associated macrophage (TAM) polarization and function (77–79), solidifying its role as a canonical M2 marker (80–82). Taken together, c-Maf emerges as a master regulator of macrophage self-renewal, differentiation, phenotypic plasticity, and anti-inflammatory responses. Its multifaceted roles underscore its potential as a therapeutic target in inflammatory diseases, cancer, and metabolic disorders (**Figure 1**).

(The figure was created with Biorender.com. Briefly, in CD8^+^ T cells, c-Maf supports TRM17 tissue residency via the ICOS-c-Maf-IL-7 axis and promotes exhaustion through the IL-27-c-Maf-PD-1 pathway. In Th2 cells, c-Maf is upregulated by IL-2/STAT5, IL-6/TCR, and STAT6, and sustains Th2 identity via SATB1-c-Maf-IL-4 signaling while suppressing IFN-γ. In Th17 cells, Sox5 and c-Maf activate RORγt; with IL-6/TGF-β, c-Maf represses IL-22 and enhances IL-10. In Tfh cells, c-Maf is induced by TGF-β/NF-κB/Thpok and promotes differentiation via CXCR5/Bcl6, Plekho1–Foxo1, and IL-21-TET2-AIM2 pathways. In B cells, the LPS/TLR4-mTOR-c-Maf axis drives IL-10 production. In ILC2s, c-Maf upregulates IL-5 and IL-13 upon allergen exposure. In macrophages, c-Maf enhances F4/80 and VCAM-1 expression. In microglia, ROS-p53 signaling downregulates c-Maf and augments inflammation).

## 3 Regulatory roles and mechanisms of c-Maf in autoimmune diseases

### 3.1 c-Maf in inflammatory bowel disease

Inflammatory bowel disease (IBD) is a chronic inflammatory disease, including Crohn’s disease (CD) and ulcerative colitis (UC). Currently available studies have implicated that the pathogenesis of IBD is associated with genetic factors, environmental influences, immune dysregulation, impaired intestinal barrier, and dysfunction of the microbiome (83). IL-10 is a key anti-inflammatory cytokine maintaining the balance between gut microbes and the immune system, which plays an important role in controlling the progression of IBD (84). Previous research has revealed that Notch/STAT3-induced c-Maf-dependent IL-10 signaling pathways are disrupted in effector CD4^+^ T cells, which leads to reduced IL-10 production and increased pro-inflammatory Th1 and Th17 cells, resulting in increased infiltration of granulocytes and myeloid cells, and worsening tissue damage in IBD (85–87). In mouse models of colitis, c-Maf deficiency inhibits the differentiation of Tr1 cells, whereas the CCR2/CCR5 dual antagonists (e.g., Cenicriviroc) restore c-Maf expression and Tr1 cell development but restrain the pro-inflammatory cytokines IFN-γ and IL-17 (23, 88). Additionally, c-Maf is essential for the differentiation and function of RORγt^+^ Tregs and CD4^+^Foxp3^+^CD69^+^ Tregs (CD69^+^ Tregs), which selectively inhibiting Th17 cells by increasing IL-10 production, while c-Maf loss leads to Tregs dysfunction, hyperactivation of Th17 cells, and severe colonic inflammation (89, 90). Overall, by governing immune cells differentiation, cytokine networks, and inflammatory responses, c-Maf emerges as a central regulator of intestinal homeostasis. Targeting c-Maf using small-molecule agonists or pathway modulation represents a promising strategy for IBD treatment, offering potential to restore immune balance and mitigate disease progression (**Table 1**).

**Table 1:** Biological roles and molecular regulatory mechanisms of c-Maf in autoimmune diseases.

Disease type	Molecular mechanisms	Biological roles	Origin of species	References
IBD	Defects in the Notch/STAT3-Blimp-1/c-Maf-IL-10 axis in CD4^+^ T cells	Driving inflammatory Th1/17 cells phenotypes and Th17 cells overactivation	Human	(85)
	Defects in Blimp-1/c-Maf-IL-10 axis	Driving Th17 cells expansion and triggers inflammatory cell infiltration	Human	(86)
	Activation of TGF-β/c-Maf pathway	Enhancing Th17 cells differentiation	Human	(87)
	Upregulation of miR-212/132 in T cells;inhibits c-Maf-IL-10 axis	Promoting Th17 cells differentiation and inhibits Tr1 differentiation	Mouse	(23)
	Downregulation of c-Maf in CCR2^+^/CCR5^+^CD4^+^T cells	Suppressing Tr1 cells generation and Th17 cells skewing	Mouse	(88)
	Silencing of STAT3/STAT5	Reducing IL-10 and c-Maf expression, impairing anti-inflammatory responses	Mouse	(90)
Autoimmune Diabetes	SUMOylation defects in c-Maf	Aberrant IL-21 activation via CBP/p300-mediated histone acetylation, accelerating β-cells destruction, promotes diabetes progression	Mouse	(91)
	Reduced Blimp-1 expression	Increased IL-21 transcription driving pathogenic T cells differentiation	Mouse	(92)
	c-Maf deficiency	Decreased IL-4/IL-5/IL-10, weakening protective Th2 cells responses	Mouse	(93)
SLE	Dysregulation of IL-21-TET2-AIM2-c-Maf pathway	Tfh cells abnormal expansion	Mouse	(94)
	STAT3-mediated suppression of c-Maf	Th17 cells differentiation promotion	Human and mouse	(95)
MS	STAT3/c-Maf-mediated enhancement of Th17 cells response suppression	Th17 cells response suppression and CNS inflammation reduction	Mouse	(96)
	Bregs promote c-Maf expression	Increased IL-10 production, improving MS	Mouse	(54)
	Elevated c-Maf expression	Increased IL-10, IL-4 and PD-1 production, alleviating disease severity	Human	(97, 98)
ITP	Elevated IL-21/IL-6/Bcl-6/c-Maf mRNA in ITP patients	Abnormal expansion of Tfh cells	Human	(99)
	Abnormal Tfh cells expansion	Elevating c-Maf expression	Human	(100)

### 3.2 c-Maf in autoimmune diabetes

Autoimmune diabetes is a progressive disorder characterized by immune-mediated destruction of pancreatic β-cells, driven by autoreactive T cells and dysregulated cytokine networks (101). Emerging evidence highlights SUMOylation, a post-translational modification involving small ubiquitin-like modifier (SUMO), as a critical regulator of inflammatory pathways contributing to disease progression (102). Research has shown that mutations in the c-Maf protein’s SUMO modification sites (KRc) in NOD mice accelerate diabetes onset by suppressing the recruitment of the repressive complex death-associated protein (DAP)/histone deacetylase 2 (HDAC2) and enhancing the activation of IL-21 as well as the recruitment of coactivators cAMP response element-binding protein-binding protein (CBP) and p300 to the IL-21 promoter’s MARE region (91). The PRDM1-encoded Blimp-1 protein inhibits IL-21 by reducing chromatin accessibility at its promoter and displacing c-Maf from the IL-21 regulatory area, thus delaying autoimmune diabetes onset in KRc-transgenic NOD mice (92). Moreover, c-Maf is essential for the differentiation of Th2 cells, which may counterbalance autoimmune aggression in transgene-induced spontaneous diabetes and virus-induced diabetes (93).

It has been well documented that c-Maf is also expressed in insulin-produced β-cells, influencing β-cells differentiation and survival (103). Therefore, c-Maf may play a vital role in the development of autoimmune diabetes by regulating β-cell-specific genes and immune interactions. Future research is warranted to explore the tissue-specific mechanisms of c-Maf in β-cells and immune cells in autoimmune diabetes, which would provide new insights into the c-Maf-targeted therapies for this disease (**Table 1**).

### 3.3 c-Maf in systemic lupus erythematosus

Systemic lupus erythematosus (SLE) is an autoimmune disease marked by the excessive activation of T cell and B cell-mediated disorders (104). Absent in melanoma 2 (AIM2), a member of the interferon-inducible HIN-200 protein family, binds to cytoplasmic double-stranded DNA (dsDNA) and forms a complex with apoptosis associated speck-like protein containing a CARD (ASC) and caspase-1 to activate the AIM2 inflammasome, which leads to the release of IL-1β and IL-18 and triggers pyroptosis (105). Increased expression of AIM2 has been demonstrated in the peripheral blood and skin lesions of SLE patients. Mechanistically, IL-21 recruits ten-eleven translocation 2 (TET2) to the AIM2 promoter, resulting in DNA demethylation and subsequent upregulation of AIM2 transcription. Furthermore, AIM2 regulates c-Maf expression, which in turn promotes IL-21 production and facilitates Tfh cells differentiation. This research demonstrates the dysregulation of the IL-21-TET2-AIM2-c-Maf signaling axis in lupus pathogenesis, highlighting its potential as a therapeutic target for SLE (94). Viral infections may exacerbate SLE by activating STAT3, which promotes IFN-α secretion and Th17 cell differentiation by suppressing c-Maf expression, leading to Th17/Tregs imbalance and autoimmune disorders (95). Recent advances in SLE research underscore the critical role of c-Maf in immune dysregulation, offering novel insights for targeted therapeutic strategies (**Table 1**).

### 3.4 c-Maf and multiple sclerosis

Multiple sclerosis (MS) is a chronic inflammatory disorder of the central nervous system (CNS) characterized by autoimmune-mediated demyelination (106). Th17 cells are significant contributors to the autoimmune inflammation and demyelination in the CNS (107, 108). The transcription factor c-Maf exerts immunomodulatory effects by binding to the promoter regions of anti-inflammatory genes, which thus suppresses the activity of Th17 cells and attenuates CNS inflammation and damages in MS (96). Bregs contribute to MS progression by boosting the production of IL-10 through the upregulation of c-Maf (54). c-Maf modulates CD8^+^ T cell function by promoting PD-1 expression and IL-10 production, while concurrently suppressing the survival of activated CD4^+^ T cells. This regulatory mechanism contributes to the containment of excessive inflammation and provides protection to the central nervous system (97). Similarly, c-Maf-high T cells acquire a regulatory phenotype characterized by IL-4 and IL-10 secretion, which helps inhibit disease progression (98). Overall, these findings suggest c-Maf as a key anti-inflammatory regulator that shapes both T cells and B cells responses, offering potential therapeutic approaches for MS (**Table 1**).

### 3.5 c-Maf and immune thrombocytopenia purpura

Immune thrombocytopenic purpura (ITP) is an antibody-mediated autoimmune disorder characterized by accelerated platelet destruction and consequent thrombocytopenia (109). Patients with ITP show significantly elevated mRNA expression of Bcl-6 and c-Maf transcription factors compared to healthy individuals, along with expansion in Tfh cells, whereas the mRNA level of c-Maf is notably reduced after treatment (99, 100). These clinical observations demonstrate that successful therapeutic intervention correlates with reduced c-Maf expression. The established association between c-Maf and Tfh cells activity strongly implicates this transcription factor in ITP pathogenesis, particularly through its regulation of Tfh-mediated autoimmune responses. These findings position c-Maf as a promising novel molecular target for ITP treatment (**Table 1**).

## 4 The potential clinical application value of c-Maf

Targeted inhibition of c-Maf has emerged as a promising therapeutic strategy in cancer treatment, with current approaches focusing on indirect suppression of c-Maf expression/activity and interception of downstream signaling cascades. In MM, hyperactivation of the MEK/ERK pathway drives c-Maf overexpression, positioning MEK inhibitors (e.g., trametinib, cobimetinib) as potential c-Maf modulators (110). The mTOR signaling pathway is involved in the regulation of c-Maf, suggesting therapeutic utility for mTOR inhibitors, such as everolimus (111). Panobinostat, a pan-deacetylase inhibitor clinically approved for MM treatment, may partially exert its anti-tumor effects through c-Maf suppression via deacetylase inhibition (112). Mechanistically, the Bcl6/Maf transcriptional complex cooperatively upregulates the expression of CXCR4 and PD-1, establishing an immunosuppressive tumor microenvironment (113). Plerixafor, a small-molecule antagonist of the CXCR4 chemokine receptor, has been utilized in treating hematologic disorders to disrupt c-Maf-mediated oncogenic signaling (114). Taken together, these findings highlight the multifaceted approaches being explored to therapeutically target c-Maf networks in cancer.

The therapeutic potential of c-Maf modulation has also shed some light on the treatment of autoimmune diseases, although significant challenges remain. However, directly targeting c-Maf is challenging due to its role as a transcription factor. Furthermore, c-Maf has a dual function in maintaining immune balance. Although the preclinical studies have demonstrated amelioration of inflammatory phenotypes through c-Maf intervention in animal models in autoimmune diseases, the exact mechanisms and long-term effectiveness still need further investigations. Future research should focus on developing highly selective c-Maf modulators with optimized safety profiles, clarifying disease-specific mechanisms of c-Maf regulation across different autoimmune pathologies, establishing robust translational frameworks for target validation, and evaluating clinical feasibility through rigorous preclinical-to-clinical pipelines. These efforts will advance c-Maf-targeted therapy from mechanistic insight to therapeutic reality in autoimmunity.

## 5 Summary and outlook

Currently available data has suggested the cytokine-STAT signaling cascade serves as a central regulator of c-Maf expression, which cooperates with lineage-defining transcription factors (e.g., GATA3, RORγt) to orchestrate immune response. This c-Maf-based interaction network plays critical roles in the regulation of cytokines production, the differentiation of immune cells, and the maintenance of immune homeostasis. Growing insights into the immunoregulatory function of c-Maf have revealed its therapeutic potential for autoimmune disorders. The unique properties of c-Maf offer multifaceted opportunities for autoimmune disease interventions. Future studies are encouraged to explore intervention strategies based on c-Maf expression patterns in specific diseases, developing combinatorial therapies that target its upstream regulators and downstream effectors and utilizing it as a dynamic biomarker through monitoring the expression and phosphorylation states to assess disease activity. As mechanistic understanding advances, c-Maf continues to emerge as a promising diagnostic and therapeutic target in immunology.
